# Regional impacts of electricity system transition in Central Europe until 2035

**DOI:** 10.1038/s41467-020-18812-y

**Published:** 2020-10-02

**Authors:** Jan-Philipp Sasse, Evelina Trutnevyte

**Affiliations:** Renewable Energy Systems, Institute for Environmental Sciences (ISE), Section of Earth and Environmental Sciences, University of Geneva, Uni Carl Vogt, Boulevard Carl Vogt 66, CH-1211, Geneva 4, Switzerland

**Keywords:** Environmental impact, Energy modelling, Renewable energy, Energy justice

## Abstract

Achieving current electricity sector targets in Central Europe (Austria, Denmark, France, Germany, Poland and Switzerland) will redistribute regional benefits and burdens at sub-national level. Limiting emerging regional inequalities would foster the implementation success. We model one hundred scenarios of electricity generation, storage and transmission for 2035 in these countries for 650 regions and quantify associated regional impacts on system costs, employment, greenhouse gas and particulate matter emissions, and land use. We highlight tradeoffs among the scenarios that minimize system costs, maximize regional equality, and maximize renewable electricity generation. Here, we show that these three aims have vastly different implementation pathways as well as associated regional impacts and cannot be optimized simultaneously. Minimizing system costs leads to spatially-concentrated impacts. Maximizing regional equality of system costs has higher, but more evenly distributed impacts. Maximizing renewable electricity generation contributes to minimizing regional inequalities, although comes at higher costs and land use impacts.

## Abstract (web summary)

Implementation of Central European electricity targets will redistribute regional benefits and burdens. Here the authors show that the aims of cost-efficiency, regional equality, and renewable electricity generation have vastly different implementation pathways, impacts, and trade-offs.

## Introduction

Climate change and other environmental impacts pose severe threats to human wellbeing in Europe and the World. To overcome these challenges, the new European Green Deal aims to reduce greenhouse gas emissions, decouple economic growth from resource use, and leave no person or place behind^1^. One of the key strategies defined in this roadmap is to maximize the deployment of renewable technologies to fully decarbonize electricity supply while ensuring equitable transition^2^. To incentivize renewable capacity deployment, most European countries have defined ambitious targets in their national energy and climate plans for the next decade^3^. Many have introduced monetary incentives, whereas significant reductions in capital cost have also made renewable technologies more financially attractive^4^. So far, these policy and market forces drove renewable capacity growth in Europe^5^.

Such an increase in renewable capacity promises many benefits for European regions, but there are unintended consequences and burdens too. Apart from limiting climate change, benefits include reduced air pollution^6,7^, chances for economic development and new employment^8,9^, additional revenues for local communities and landowners^10,11^, and decreased dependence from imported fossil fuels^12^. On the other hand, burdens include increased electricity system costs^13,14^, adverse impacts on ecosystems and wildlife^15^, visual and sound annoyance^16,17^, threats to employment due to phase-outs of fossil fuels^18,19^, land use conflicts^20^, and decreased property values^9,21^. Until now, renewable capacity was being unevenly deployed across European regions^22,23^, indicating that the associated regional impacts are unevenly distributed as well. Thus, the electricity sector transition risks creating new regional winners and losers. Finding ways to anticipate and minimize emerging regional inequalities can foster the implementation success of the transition.

A way to investigate regional impacts of this transition is to model spatially explicit future scenarios of the electricity sector. Despite the abundance of European electricity sector models and scenarios, there are only few that study the associated regional impacts. Several studies so far have focused on technical and economic aspects, such as the least-cost allocation of renewable capacity^13^, the weather effects on costs and renewable generation^14,24^, or the regional economic impacts^25^. Even fewer models exist that consider regional impacts from the lens of regional equality. Some have modeled the regionally equitable allocation of solar PV and wind capacity in Germany^26^, or of all renewable capacity in Switzerland^27^. Others have modeled regional equality of electricity access and solar PV deployment in sub-Saharan Africa^28,29^. While providing initial first steps, existing studies do not constitute a more holistic quantitative picture on regional impacts and trade-offs associated with the electricity sector transition, such as regional employment or land use impacts. Existing studies on spatial equality also often neglect technical system feasibility in terms of hourly operation, transmission, and storage.

Here, we quantify the regional impacts of electricity sector transition in 2035 in six countries of Central Europe (Austria, Denmark, France, Germany, Poland, and Switzerland) at the spatial level of 650 NUTS-3 regions^30^ (see “Methods”). We soft link two optimization models called EXPANSE^27,31^ and PyPSA^32^. Using modeling to generate alternatives (MGA)^27,33,34^, an uncertainty technique to search for cost-optimal and near-optimal solutions in optimization models^34,35^, we compute 100 spatially explicit scenarios of electricity generation, storage, and transmission for the countries to achieve their current national electricity targets. From these 100 MGA scenarios, we estimate the associated regional impacts^36^ regarding system costs, employment, greenhouse gas emissions, particulate matter emissions, and land use. We evaluate how equally these impacts are distributed across NUTS-3 spatial level regions, where regional equality is measured using the Gini index^37^ (see “Methods”). We analyze patterns in these 100 spatially explicit MGA scenarios, from which we select three distinct scenarios to highlight trade-offs: scenarios with minimum system costs, with maximum regional equality of system costs, and with maximum renewable electricity generation. We compare these scenarios with a fourth distinct scenario of frozen generation capacity, where generation capacities in 2035 remain as in 2018. We show that these three aims have vastly different implementation pathways as well as associated regional impacts and cannot be optimized simultaneously. Minimizing system costs leads to spatially concentrated impacts. Maximizing regional equality of system costs has higher, but more evenly distributed impacts. Maximizing renewable electricity generation contributes to minimizing regional inequalities, although comes at higher costs and land use impacts.

## Results

### Overall scenario ranges of system infrastructure and impacts

**Fig. 1:**
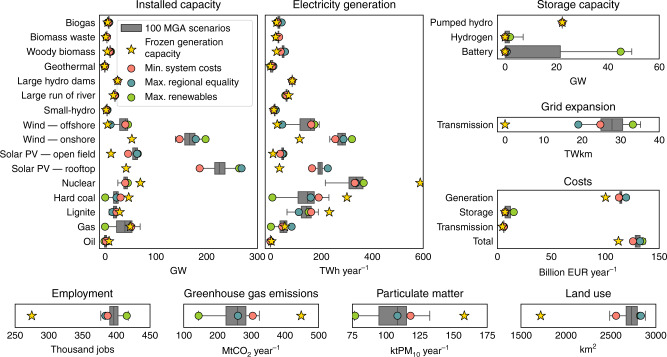
Scenarios of electricity generation in 2035. The figure shows technology-specific generation, storage, and transmission, as well as associated costs and regional impacts for the scenario of frozen generation capacity and the 100 modeling to generate alternatives (MGA) scenarios. Amongst the 100 MGA scenarios, we highlight the scenarios of minimum system costs, maximum regional equality of system costs, and maximum renewable electricity generation. Box plot elements are: center line, median; box limits, upper and lower quartiles; whiskers, minimum and maximum values. MGA scenarios are generated with a slack of 20% above total levelized electricity generation costs of the cost-optimal solution in the EXPANSE model and assuming that each country meets its national electricity generation targets for 2035 (see “Methods”). Total system costs include annual capital and variable costs (i.e., fuel, operation, and maintenance) for electricity generation, storage, and transmission. Employment impact includes annual direct employment for electricity generation and storage, including jobs in construction, installation, operation, maintenance, and decommissioning. For biomass, coal, and lignite, we additionally include jobs in fuel extraction and transport. Greenhouse gas emissions impact includes annual direct greenhouse gas emissions from fuel combustion for electricity generation. Particulate matter impact includes annual direct particulate matter emissions from fuel combustion for electricity generation. Land use impact includes direct land use for electricity generation. Source data are provided in Supplementary Data 1.

In these MGA scenarios, electricity generation varies considerably in the six countries for wind, solar PV, nuclear, and fossil fuels, indicating flexibility in implementing the national electricity targets, but it is relatively constant for biomass, geothermal, and hydropower (Fig. 1). Compared to the scenario with frozen generation capacity (without national electricity targets), the other 100 MGA scenarios (with targets) have higher electricity generation from offshore wind (33–192 TWh year^−1^ compared to 27  TWh year^−1^), onshore wind (233–320 TWh year^−1^ compared to 115 TWh year^−1^), open-field solar PV (37–53 TWh year^−1^ compared to 10 TWh year^−1^), and rooftop solar PV (164–231 TWh year^−1^ compared to 36 TWh year^−1^). In contrast, they have lower electricity generation from nuclear (216–371 TWh year^−1^ compared to 588 TWh year^−1^), hard coal (2–230 TWh year^−1^ compared to 300 TWh year^−1^), and lignite (65–188 TWh year^−1^ compared to 231 TWh year^−1^). Gas-based electricity generation is between 0 and 90 TWh year^−1^ compared to 57 TWh year^−1^. Despite the definite growth of solar PV and wind power, there is much leeway in this near-optimal space of 100 MGA scenarios for these technologies. Large reductions in nuclear and coal-based electricity generation are apparent to reach the national electricity targets. Gas-based electricity generation either increases or decreases depending on the other generation capacities.

The four distinct scenarios differ in terms of technology-specific electricity generation. The scenario with minimum system costs is characterized by relatively high centralized electricity generation from hard coal (190 TWh year^−1^), lignite (160 TWh year^−1^), offshore wind (161 TWh year^−1^), large hydropower dams (86 TWh year^−1^), large run-of river (63 TWh year^−1^), and biomass waste (35 TWh year^−1^), suggesting that these technologies improve cost efficiency. The scenario with maximum regional equality of system costs has comparatively high decentralized electricity generation: onshore wind (287 TWh year^−1^), rooftop solar PV (226 TWh year^−1^), open-field solar PV (48 TWh year^−1^), woody biomass (57 TWh year^−1^), biogas (45 TWh year^−1^), small hydropower (22 TWh year^−1^), geothermal (12 TWh year^−1^), and gas (84 TWh year^−1^). Due to their decentralized nature, these technologies improve regional equality. Nuclear electricity generation is 332 TWh year^−1^ and does not differ much between the scenarios of cost efficiency and regional equality. The scenario with maximum renewable electricity generation has high electricity generation from renewable technologies (1037 TWh year^−1^), nuclear plants (366 TWh year^−1^), and above-average lignite-based generation (150 TWh year^−1^). In this case, nuclear and fossil fuel electricity enable renewable electricity integration by keeping total system costs within the near-optimal range in 2035. Lignite rather than gas is selected by the model due to lower generation costs, which results in cost savings even with more renewable electricity. All distinct scenarios except for frozen generation capacity forego oil electricity generation (<1 TWh year^−1^).

Storage and transmission capacities vary considerably across 100 MGA scenarios, with up to 7 GW of additional hydrogen and 50 GW of additional battery storage capacity as compared to 2018. The scenarios with frozen generation capacity and minimum system costs have no additional storage capacity, whereas the scenario with maximum regional equality has 675 MW battery and 75 MW hydrogen storage. In comparison, the scenario with maximum renewable electricity generation requires very high amounts of 45 GW battery and 2 GW hydrogen storage. Storage capacity is thus associated with renewable electricity integration and, to a lesser extent, regional equality of system costs. To balance more intermittent renewable electricity, the scenario with maximum renewable electricity generation requires very high transmission capacity expansion of 33 TWkm in total too. In comparison to this scenario, the scenario with minimum system costs requires 25 TWkm and therefore 24% less transmission capacity expansion, while the scenario with maximum regional equality requires only 19 TWkm and therefore 42% less. Thus, transmission capacity expansion is associated with renewable electricity integration and, to a lesser extent, cost efficiency.

Electricity generation, storage, transmission, and total system costs are consistently higher for all 100 MGA scenarios compared to the scenario with frozen generation capacity (that has generation capacity of 2018 without the six countries reaching their national electricity targets). For MGA scenarios, these costs vary between 112 and 120 billion EUR year^−1^ for generation, 7 and 15 billion EUR year^−1^ for storage, 5 and 6 billion EUR year^−1^ for transmission, and 125 and 137 billion EUR year^−1^ for the total system costs. For frozen generation capacity, these costs are 100 billion EUR year^−1^, 7 billion EUR year^−1^, 5 billion EUR year^−1^, and 112 billion EUR year^−1^, respectively. The scenario with minimum system costs has low generation, storage, and total system costs at 112 billion EUR year^−1^, 7 billion EUR year^−1^, and 125 billion EUR year^−1^, but high transmission costs at 6 billion EUR year^−1^. The scenario with maximum regional equality of system costs has high generation and total system costs at 119 billion EUR year^−1^ and 132 billion EUR year^−1^, but low storage and transmission costs at 7 billion EUR year^−1^ and 5 billion EUR year^−1^. The scenario with maximum renewable electricity has low generation costs of 113 billion EUR year^−1^, but high storage, transmission, and total system costs at 15 billion EUR year^−1^, 6 billion EUR year^−1^, and 134 billion EUR year^−1^. Renewable electricity integration, rather than regional equality of system costs, increases total system costs.

Direct employment for all MGA scenarios is 376–417 thousand jobs, attributed mainly to increased solar PV and wind capacities. In comparison, the scenario with frozen generation capacity has far less employment with 274 thousand jobs, attributed mainly to existing nuclear and coal capacities. The scenarios with minimum system costs and maximum regional equality have similar employment of 387 and 384 thousand jobs, respectively. The scenario with maximum renewable electricity generation has high employment with 415 thousand jobs. Overall, national electricity targets lead to an increase in employment in the electricity sector. Maximizing renewable generation, rather than minimizing system costs or maximizing regional equality of system costs, leads to increases in employment.

In terms of environmental impacts, direct greenhouse gas emissions from electricity generation for all MGA scenarios is between 144 and 324 MtCO_2-eq_ year^−1^, which is much lower than 448 MtCO_2-eq_ year^−1^ for the scenario with frozen generation capacity. Compared to all MGA scenarios, the scenario with minimum system costs has high greenhouse gas emissions of 304 MtCO_2-eq_ year^−1^. In comparison, the scenario with maximum regional equality has average emissions of 260 MtCO_2-eq_ year^−1^, and the scenario with maximum renewable electricity generation has the lowest emissions of 144 MtCO_2-eq_ year^−1^. Direct particulate matter emissions from electricity generation are between 76 and 122 ktPM_10-eq_ year^−1^ for all MGA scenarios, which is far less than in the scenario with frozen generation capacity (135 ktPM_10-eq_ year^−1^). For scenarios with minimum system costs and maximum regional equality, particulate matter emissions are 118 and 100 ktPM_10-eq_ year^−1^, respectively. The scenario with maximum renewable electricity generation has low emissions of 77 ktPM_10-eq_ year^−1^. Therefore, to reduce greenhouse gas and particulate matter emissions, the priority is to maximize renewable electricity generation and, to a lesser extent, regional equality of system costs, instead of minimizing total system costs. The main reason why regional equality enables lower greenhouse gas and particulate matter emissions than cost efficiency is twofold. First, regional equality discourages spatial concentration of large centralized coal generation with high emissions. Second, regional equality encourages a regionally even distribution of generation capacities and this is mostly achieved with additional renewable generation capacities with low direct emissions.

Direct land use for electricity generation infrastructures for all MGA scenarios is between 2486 and 2882 km^2^, which is far higher than in the scenario with frozen generation capacity with 1719 km^2^. The increase in land use is mostly due to higher onshore wind, open-field solar PV, woody biomass, and biogas capacities. The scenario with minimum system costs has low land use of 2557 km^2^ compared to the scenario with maximum regional equality with 2833 km^2^ due to a more cost efficient spatial allocation of wind and solar PV, and higher fossil fuel capacities, which require less land. The scenario with maximum renewable electricity generation has relatively high land use of 2824 km^2^. Overall, high land use is attributed to the focus on regional equality of system costs and renewable electricity generation, whereas less extensive land use is attributed to cost efficiency.

### Regional distribution of electricity system infrastructure

**Fig. 2:**
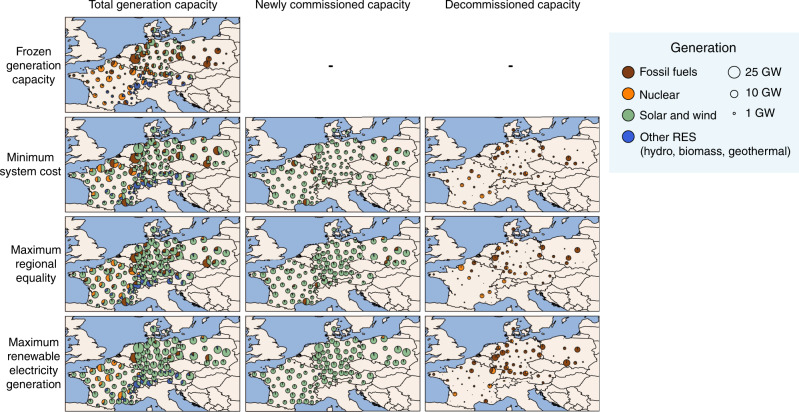
Regional differences in electricity generation capacity in 2035. The figure depicts total, newly commissioned, and decommissioned electricity generation capacity as compared to 2018 at each of the 100 grid nodes for the four distinct scenarios of frozen generation capacity, minimum system costs, maximum regional equality of system costs, and maximum renewable electricity generation. In the scenarios of minimum system costs, maximum regional equality of system costs, and maximum renewable electricity generation, each country meets its national electricity generation targets for 2035 (see “Methods”). Generation capacities are aggregated to the four categories for visualization purposes. Fossil fuels category includes gas, hard coal, lignite, and oil. Solar and wind category includes onshore wind, offshore wind, rooftop solar PV, and open-field solar PV. Other renewable energy sources (RES) category includes hydropower (including large dams, large run-of-river, and small hydropower), biomass (including biogas, biomass waste, and woody biomass), and geothermal power.

**Fig. 3:**
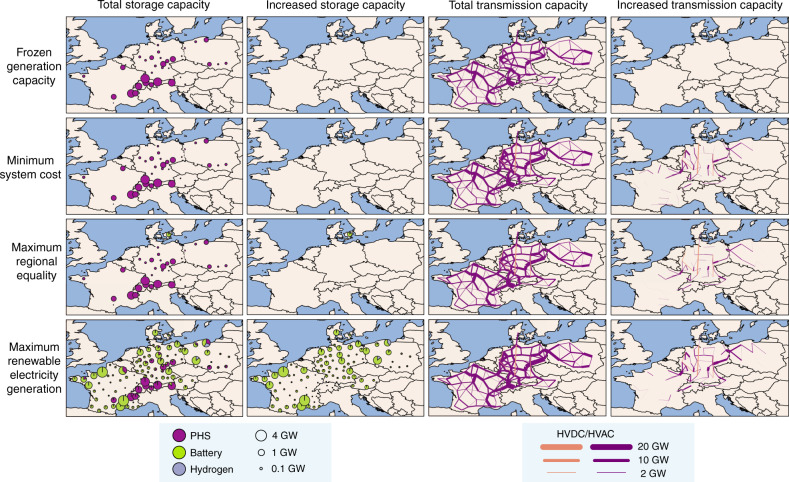
Regional changes in storage and transmission capacity in 2035. The figure depicts total storage and transmission capacity as well as increases in this capacity at each of the 100 grid nodes as compared to 2018 for the four distinct scenarios of frozen generation capacity, minimum system costs, maximum regional equality of system costs, and maximum renewable electricity generation. In the scenarios of minimum system cost, maximum regional equality of system costs, and maximum renewable electricity generation, each country meets its national electricity generation targets for 2035 (see “Methods”). PHS pumped hydro storage; HVDC high voltage direct current transmission line; HVAC high voltage alternating current transmission line.

The scenario with frozen generation capacity that represents generation in 2018 (Fig. 2) has high hydropower capacities in Austria and Switzerland; a mix of biomass, fossil fuels, solar PV, and wind capacities in Denmark and Germany; high nuclear capacities in France; and high fossil fuel capacities (mostly coal and lignite) in Poland (see Supplementary Fig. 1 for electricity generation). Existing regional generation, storage, and transmission capacities provide enough flexible electricity for all regions, even with the projected 8% overall increase in electricity demand for 2035 (see “Methods”). Storage capacity for this scenario is entirely in the form of pumped hydropower and located mostly in west Austria, south-east France, and in Switzerland (Fig. 3). With these generation and storage infrastructures, transmission capacities are high in north and south-east France, south-west and east Germany, and north-east Switzerland.

The scenario with minimum system costs is characterized by relatively spatially concentrated generation capacities. Compared to all MGA scenarios, this scenario has high offshore wind capacities, mostly off the coasts of France and Germany. High onshore wind capacities are near the coasts, across Poland and in east Austria, due to high wind speeds and low generation costs. High solar PV capacities are located in south France and Switzerland due to high solar irradiation and low generation costs. Less nuclear generation in France and coal-based generation in Germany and Poland are required as countries meet their national electricity targets. More gas-based generation in France, Germany, Poland, and Switzerland are required to balance intermittent renewable electricity. Storage capacities do not need to be extended in this scenario, suggesting that existing pumped hydropower and fossil fuel capacities provide sufficient flexibility. However, significant transmission capacity expansions are needed in all countries, except for Denmark. Spatially concentrated generation thus improves cost efficiency.

The scenario with maximum regional equality of system costs is characterized by a more regionally equal distribution of generation capacity. This is achieved by increasing solar PV and wind capacities more evenly in all regions, in addition to keeping some existing nuclear and fossil fuel capacities. Onshore wind and solar PV are allocated in less windy and sunny regions with higher generation costs. Offshore wind capacities are low as they increase regional inequality of system costs. This scenario has lower coal-based generation in Germany and Poland, but higher gas-based generation in France, Poland, and Switzerland. Nuclear capacity is completely decommissioned in Germany and Switzerland, but most is kept in France. Additional storage capacity is not needed in almost all regions, except for battery and hydrogen storage in Denmark due to higher residual loads and low transmission flexibility. Transmission expansion is needed to a lesser extent in all countries than in the scenario with minimum system costs. A more regionally equal distribution of system costs requires less transmission capacity and more storage capacity as compared to the scenario with minimum system costs. Overall, decentralized generation and storage improve regional equality.

The scenario with maximum renewable electricity generation is characterized by a substantial increase in solar PV and wind capacities in all regions and a substantial decrease in fossil fuel capacities in Austria, Denmark, France, Germany, and Poland. Although low, some remaining fossil fuel capacities (mostly lignite) are located in the west and east of Germany and south Poland. France and Switzerland still have relatively high nuclear capacity. Due to the lack of fossil fuel capacities to balance intermittent electricity, high battery storage capacities are required in most regions, in addition to high transmission capacities. Highest storage capacities are installed near the coasts of Denmark, France, Germany, and Poland, and between Austria and Germany, and between Germany and Poland. Switzerland requires almost no battery storage, due to sufficient pumped hydropower. Hydrogen storage is installed in north and south Poland. Transmission capacity expansion is highest between France and Germany, and Germany and Poland. In sum, renewable and nuclear generation, as well as storage and transmission improve the aim of maximizing renewable electricity integration.

### Regional impacts of electricity system infrastructure

**Fig. 4:**
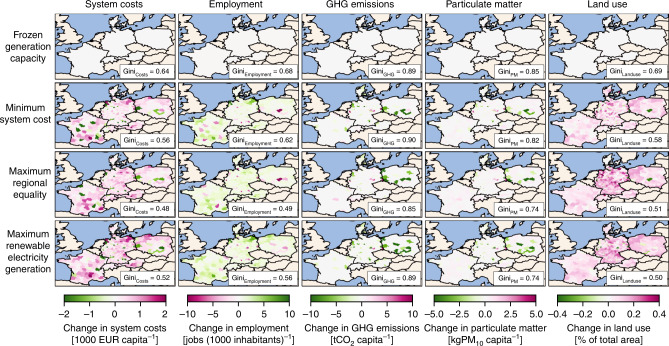
Regional impacts from generation, storage, and transmission in 2035. The values show changes in system costs, employment, greenhouse gas (GHG) emissions, particulate matter emissions, and land use per NUTS-3 region for the four distinct scenarios of frozen generation capacity, minimum system costs, maximum regional equality of system costs, and maximum renewable electricity generation, as compared to the scenario with frozen generation capacity that represents the electricity system infrastructure from 2018. A Gini index of 1.0 indicates perfect regional inequality and a Gini index of 0.0 indicates perfect regional equality. In the scenarios of minimum system cost, maximum regional equality of system costs, and maximum renewable electricity generation, each country meets its national electricity generation targets for 2035 (see “Methods”). System costs include annual capital and variable costs (i.e., fuel, operation, and maintenance) for electricity generation, storage, and transmission. Employment impact includes annual direct employment for electricity generation and storage, including jobs in construction, installation, operation, maintenance, and decommissioning. For biomass, coal, and lignite, we additionally include jobs in fuel extraction and transport. Greenhouse gas emissions impact includes annual direct greenhouse gas emissions from fuel combustion for electricity generation. Particulate matter impact includes annual direct particulate matter emissions from fuel combustion for electricity generation. Land use impact includes direct land use for electricity generation. Source data are provided in Supplementary Data 1.

When national electricity targets are met, some NUTS-3 regions are more impacted than others as compared to the scenario with frozen generation capacity that represents 2018, as shown in Fig. 4 (see Supplementary Fig. 2 for total impacts). The scenario with minimum system costs has a high increase in system costs per capita near the coasts, such as in Weser-Ems (Germany) and West Pomerania (Poland) due to high wind speeds and land availability. Substantial decreases in system costs are in regions with currently high nuclear and fossil fuel capacities in France, Germany, and Poland. The scenario with maximum regional equality has more regionally even and less extreme increases in system costs. Regional system costs increase toward inland regions due to more onshore wind and solar PV. There are fewer regions with decreased system costs, especially in France. One exception is the region of Spree-Neisse (Germany) that decommissions coal capacities. The scenario of maximum renewable electricity generation has a relatively high increase in system costs, both near the coasts and toward the inland due to high solar PV, wind, storage, and transmission capacities. Similar to all other distinct scenarios, we find a net decrease in system costs in regions with currently high coal capacities. In contrast, there are fewer changes in system costs in regions with currently high nuclear capacities. For all distinct scenarios, we find minor changes in system costs per capita in Denmark and Switzerland.

Regarding employment, the scenario with minimum system costs has the highest net increase in jobs in Austria and near the coasts of France, Germany, and Poland due to high wind capacity. The highest increase of up to 53 jobs per 1000 inhabitants is in the regions of Weser-Ems (Germany), West Pomerania (Poland), and Languedoc-Roussillon (France) as a result of high wind and solar PV capacities. The most significant net decrease in jobs is in regions with currently high nuclear and coal capacities in France, Germany, and Poland. The scenario with maximum regional equality has a more evenly distributed increase in jobs, where some regions in France, like Upper Normandy, with less jobs from decommissioned nuclear capacities have only minor net changes due to additional jobs from solar PV and wind. The scenario with maximum renewable electricity generation combines the spatial patterns from the two former scenarios. In this scenario, high increases in jobs occur near the coasts of Weser-Ems (Germany), West Pomerania (Poland), and Mecklenburg-Vorpommern (Germany), but also in inland regions of Salzburg (Austria) and Thuringia (Germany). We find a lower decrease in jobs in regions with currently high employment in coal-based generation due to additional employment in solar PV and wind generation. For example, the coal region of Piotrków County (Poland) has no net job losses for the scenario with maximum renewable electricity generation, as compared to ten lost jobs per 1000 inhabitants in the scenarios with minimum system costs and maximum regional equality.

For direct greenhouse gas and particulate matter emissions from electricity generation as well as land use, we observe similar patterns across all scenarios. The scenarios with minimum system costs, maximum regional equality, and maximum renewable electricity generation that all achieve national electricity targets for 2035 have decreases in both types of emissions in regions with currently high coal-based generation, especially in Spree-Neisse (Germany), Radomski and Sosnowiecki (Poland). Some regions have increased emissions, such as Neuss and Görlitz (Germany), due to higher gas-based electricity generation. For this reason, the scenario with maximum regional equality has a slight increase in emissions in Bouches-du-Rhône (France) and Zurich (Switzerland). The scenario of maximum renewable electricity generation has the most substantial decrease in emissions and most regions are benefitting from these impacts. Regarding the land use for electricity generation, we find an increase for most regions and all distinct scenarios that achieve national electricity targets. This is due to higher onshore wind, open-field solar PV, biogas, and woody biomass capacities. The scenario with minimum system costs has increased land use near the coasts of Denmark, Germany, and Poland, at the borders of Germany and Poland, in east Austria and south France. In comparison, the scenario with maximum regional equality has even higher land use due to higher renewable generation, especially in Weser-Ems (Germany), which amounts to 0.65% of total regional land. The scenario with maximum renewable electricity generation has similarly high land use, but it has fewer regions with severe increase as the land use is more regionally even. Only some coal regions in Germany and Poland have a net decrease for all distinct scenarios, including Spree-Neisse and Saalekreis (Germany) and Koniński (Poland).

### Regional equality and trade-offs

**Fig. 5:**
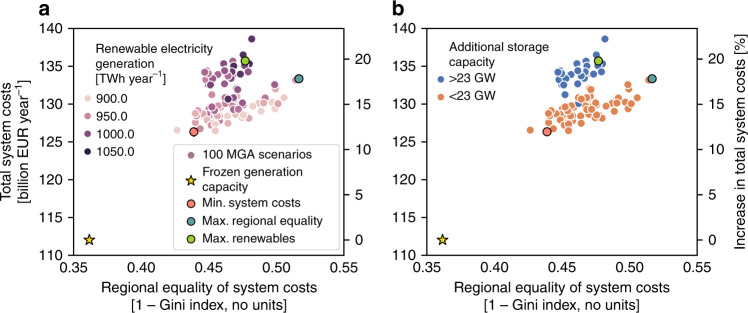
Trade-off between total system costs and regional equality in 2035. Values show total system costs and regional equality of system costs per capita for the scenario of frozen generation capacity and 100 modeling to generate alternatives (MGA) scenarios. Amongst the 100 MGA scenarios, we highlight the scenarios of minimum system costs, maximum regional equality of system costs, and maximum renewable electricity generation. Percentage values on the second *y*-axis are relative to the scenario of frozen generation capacity. **a** Colors represent the trade-off with the extent of renewable electricity generation. **b** Colors represent the trade-off with additionally required storage capacity, where the cut-off value of 23 GW additional capacity was selected for visual purposes. MGA scenarios are generated with a slack of 20% above total levelized electricity generation costs of the cost-optimal solution in EXPANSE and assuming that each country meets its national electricity generation targets for 2035 (see “Methods”). Source data are provided in Supplementary Data 1.

When analyzing all 100 MGA scenarios, we find a significant trade-off between minimizing total system costs, maximizing regional equality of system costs, and maximizing renewable electricity generation (Fig. 5). These three aims thus cannot be optimized simultaneously. Compared to the scenario with minimum system costs, the scenario with maximum regional equality has 18% higher regional equality of system costs per capita (52% compared to 44%) and 4% higher renewable electricity generation (928 TWh year^−1^ compared to 892 TWh year^−1^). As a trade-off, it has 6% higher total system costs (132 billion EUR year^−1^ compared to 125 billion EUR year^−1^). Thus, regional equality improves renewable electricity integration, but decreases cost efficiency. Compared to the scenario with maximum regional equality, the scenario with maximum renewable electricity generation has 12% higher renewable electricity generation (1037 TWh year^−1^ compared to 928 TWh year^−1^), but it also has 2% higher total system costs (134 billion EUR year^−1^ compared to 132 billion EUR year^−1^) and 8% lower regional equality of system costs (48% compared to 52%). Therefore, renewable electricity generation leads to medium regional equality due to spatial constraints of allocating renewable generation capacities, and relatively lower cost efficiency due to high investment needs in renewable generation and storage capacities.

**Fig. 6:**
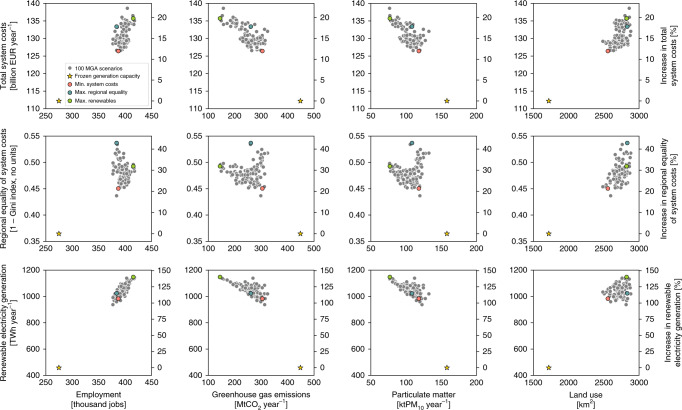
Costs, regional equality, renewable electricity generation, and impacts in 2035. Trade-off between regional impacts and total system costs, regional equality of system costs per capita, and renewable electricity generation. Regional impacts include employment, greenhouse gas and particulate matter emissions, and land use. Gray scatter points depict the 100 MGA scenarios. We additionally highlight the four distinct scenarios of frozen generation capacity, minimum system costs, maximum regional equality of system costs, and maximum renewable electricity generation. Percentage values on the second *y*-axis are relative to the frozen generation scenario. MGA scenarios are generated with a slack of 20% above total levelized electricity generation costs of the cost-optimal solution in EXPANSE model and assuming that each country meets its national electricity generation targets for 2035 (see “Methods”). Source data are provided in Supplementary Data 1.

The trade-offs between total system costs, regional equality of system costs, renewable electricity generation, and the four other regional impacts are shown in Fig. 6. Compared to the scenario with minimum system costs, the scenario with maximum regional equality has 14% lower greenhouse gas emissions, 8% lower particulate matter emissions, 11% higher land use, and 1% lower employment. Compared to the scenario with maximum regional equality, the scenario with maximum renewable electricity generation has 45% lower greenhouse gas emissions, 29% lower particulate matter emissions, similar land use, and 8% higher employment. These results suggest that some aims improve only certain regional impacts. Compared to all MGA scenarios, the aim of minimizing total system costs enables relatively lower land use, but also lower employment, as well as higher greenhouse gas and particulate matter emissions. By this comparison, the aim of maximizing regional equality of system costs means medium levels of greenhouse gas and particulate matter emissions, but higher land use and lower employment. The aim of maximizing renewable electricity generation encourages higher employment, and lower greenhouse gas and particulate matter emissions, but also higher land use.

**Fig. 7:**
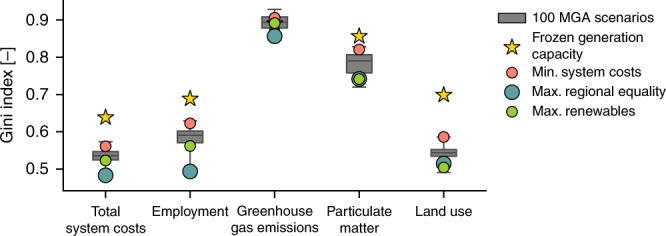
Gini index of regional impacts. Box plots (in gray) show the Gini index for the spatial distribution of regional impacts on system costs, employment, greenhouse gas emissions, particulate matter emissions, and land use for 100 modeling to generate alternatives (MGA) scenarios. In addition, we highlight the Gini index of regional impacts for the four distinct scenarios of frozen generation capacity, minimum system costs, maximum regional equality of system costs, and maximum renewable electricity generation. A Gini index of 1.0 indicates perfect regional inequality and a Gini index of 0.0 indicates perfect regional equality. Box plot elements: center line, median; box limits, upper and lower quartiles; whiskers, minimum and maximum values. MGA scenarios are generated with a slack of 20% above total levelized electricity generation costs of the cost-optimal solution in EXPANSE and assuming that each country meets its national electricity generation targets for 2035 (see “Methods”). Source data are provided in Supplementary Data 1.

We also evaluate how even all these impacts are distributed across the sub-national regions (Fig. 7), which we measure with the Gini index (see “Methods”). Regarding system costs per capita, the scenario with frozen generation capacity has the least even distribution of system costs. Amongst all 100 MGA scenarios that reach national electricity targets, the scenario with minimum system costs has a rather uneven distribution of system costs. In contrast, the scenario with maximum regional equality has the most even distribution, whereas the scenario with maximum renewable electricity generation has above-average equality too. Regarding employment, the scenario with frozen generation capacity has the most uneven regional distribution of jobs per 1000 inhabitants and this is improved in the scenarios with minimum system costs or especially maximum regional equality. The scenario with maximum renewable electricity generation has an above-average equality in terms of employment impacts too. Overall, employment impacts are more unevenly distributed across regions compared to system costs, because technology-specific generation costs (main component of system costs) vary less than jobs. For example, levelized electricity generation costs of wind and solar PV in 2035 are similar to those of fossil fuels, but the associated jobs per MWh are up to five times lower. Therefore, regional differences in electricity generation lead to more extreme regional differences in jobs than in system costs.

Regarding environmental impacts, greenhouse gas emissions are much more unevenly distributed across regions. The scenario with frozen generation capacity has a medium even distribution of greenhouse gas emissions and the scenario with minimum system costs is less even due to a substantial decrease of emissions in some regions and minor changes in others. The greenhouse gas emissions are lower and slightly more evenly distributed for the scenarios with maximum regional equality and maximum renewable electricity generation. Particulate matter emissions for all MGA scenarios are lower and more evenly distributed than for the scenario with frozen generation capacity due to decreased emissions in coal regions. The scenario with minimum system costs has an uneven distribution due to high emissions in few coal regions. The scenarios with maximum regional equality and maximum renewable electricity generation have a more even distribution of low particulate matter emissions. Regarding land use, the scenario with frozen generation capacity has the most uneven distribution and land use for all MGA scenarios that achieve country targets is higher, yet more evenly distributed. The scenario with minimum system costs has a relatively uneven distribution, whereas the most even distribution is in the scenario with maximum renewable electricity generation, followed by the scenario with maximum regional equality. Overall, we find that maximizing regional equality of system costs and renewable electricity generation leads to rather high regional equality of employment, greenhouse gas and particulate matter emissions, and land use. Minimizing system costs leads to rather low regional equality of all impacts, although regional equality is higher than if national electricity targets are not met.

## Discussion

This analysis provides an assessment at NUTS-3 level of impacts and regional equality associated with reaching the Central European targets for the electricity sector in 2035. Even for this short- to mid-term horizon, our results already show large changes in the required generation, storage, and transmission to reach these targets. Regional impacts in terms of system costs, employment, greenhouse gas and particulate matter emissions, and land use are mostly driven by changes in generation capacities of few technologies, such as solar PV, wind, nuclear, coal, and gas. Hydropower, biomass, and geothermal capacities do not change as much and have lower regional impacts. We find that the three aims of minimizing system costs, maximizing regional equality, and maximizing renewable electricity generation have vastly different regional implementation pathways and cannot be reached simultaneously. Minimizing system costs requires high centralized generation capacities of coal, offshore wind, large hydropower, and biomass waste, and high transmission capacities. Maximizing regional equality of system costs requires high decentralized renewable and gas-based generation capacities, and additional storage capacities. Maximizing renewable electricity generation requires high renewable and nuclear generation capacities, and very high storage and transmission capacities. We find that a complete phase-out of lignite and nuclear by 2035 is not feasible within our modeled cost limits, while a phase-out of hard coal, gas, and oil is. Accounting for higher cost deviations might still not allow for a complete phase-out of lignite and nuclear generation though, especially in Poland and France, due to limited technology build rates. Compared to 2018, Central European electricity targets of 2035 increase regional equality of system costs by an additional 18–43% and increase renewable electricity generation by an additional 97–140%. System costs increase by 12–22%, but such cost increases could still be acceptable for environmental or social goals.

Achieving Central European electricity targets would lead to significant net increases in system costs, employment, and land use, and significant net decreases in greenhouse gas and particulate matter emissions. These net regional impacts differ for different aims that we analyzed. Minimizing total system costs would lead to relatively low land use, but also low increase in employment and low reductions in greenhouse gas and particulate matter emissions. If system costs are distributed most evenly, emissions would decrease further due to higher renewable generation, but such a distribution would have a detrimental impact on land use. This would not have higher employment, as it would encourage job-poor solar PV, onshore wind and gas-based generation, and discourage job-rich offshore wind and coal-based generation. Maximizing renewable electricity generation would not further increase land use, as it would encourage nuclear, rooftop solar PV, and offshore wind capacities with low land use and cost efficiently allocate open-field solar PV and onshore wind capacities. It would lead to high employment, and lowest greenhouse gas emissions, and particulate matter.

These three aims of minimizing costs, maximizing equality, and maximizing renewable generation have different spatial patterns in terms of regional generation, storage, and transmission capacities. Minimizing system costs encourages a spatial concentration of wind capacities in regions with high wind speeds and low generation costs, such as near the coasts of Denmark, France, Germany, and Poland, and in the east of Austria. Similarly, it encourages a spatial concentration of solar PV capacities in regions with high solar radiation and low generation costs, such as the south of France and Switzerland. It is cost efficient to keep most nuclear capacities in France, and coal capacities in Germany and Poland. Instead of investing in battery and hydrogen storage, it is cost efficient to extend transmission capacities between countries and near the coasts to balance intermittent electricity. In contrast, maximizing regional equality of system costs encourages a more even distribution of generation capacities by installing less offshore and onshore wind capacities near the coasts, and more onshore wind and solar PV capacities toward inland regions. It encourages less coal capacities in Germany and Poland, but higher decentralized gas-based generation in France, Poland, and Switzerland. It requires less transmission capacities but higher battery storage capacities. In comparison, maximizing renewable electricity generation encourages high renewable capacities both near the coasts and toward the inland. Even though coal capacities are low, some are still required in Germany and Poland to keep costs within modeled limits. High transmission and storage capacities are required near the coasts, between countries and close to demand to balance the increase in intermittent electricity. Only Switzerland requires low battery storage capacity due to sufficient pumped hydropower capacity. Overall, we find that transmission capacity expansion cannot be avoided for all scenarios and regions. In contrast, additional storage capacity can be avoided, if sufficient transmission and fossil fuel capacities exist.

The assessed three aims have different spatial patterns in terms of regional impacts as well. Minimizing system costs encourages a spatial concentration of increased system costs near the coasts and in the south, and decreased system costs in nuclear regions in France, and coal regions in Germany, and Poland. Such changes in regional system costs are linked to changes in regional employment. The windiest and sunniest regions have the highest increase in jobs, while nuclear and coal regions have the highest decrease in jobs. As a consolation, coal regions would benefit from improved human health from reduced particulate matter emissions and emit less greenhouse gas emissions. Land use would increase substantially in most regions, especially near the coasts of Germany and Poland, or the south of France, and only coal regions would benefit from reduced land use. In comparison, encouraging a regionally even distribution of system costs would lead to less severe increases in system costs in coastal regions, and a more even increase across inland regions. Such a distribution would encourage a more even distribution of additional jobs across all regions and would further reduce greenhouse gas and particulate matter emissions in most regions. In comparison, maximizing renewable electricity generation would have even higher system costs near the coasts and in the south, but also in less windy and sunny regions. Such an aim would have the highest overall increase in jobs, but also very high land use. Coal regions of Germany and Poland would benefit from reductions in greenhouse gas and particulate matter emissions, and land use. For all three aims, positive impacts occur more in current coal regions rather than nuclear regions. More regions are impacted by changes in system costs, employment, and land use than by changes in particulate matter and greenhouse gas emissions.

By assessing the spatial distribution of regional impacts with the Gini index, we observe clear differences in terms of regional equalities and inequalities between the three aims. Minimizing system costs leads to rather spatially concentrated regional impacts in terms of system costs, employment, greenhouse gas and particulate matter emissions, and land use, and therefore encourages regional inequality. Maximizing regional equality of system costs leads to a rather even distribution of all assessed impacts and therefore encourages regional equalities. Maximizing renewable electricity generation contributes to minimizing regional inequalities, although at higher total system costs and land use impacts. This result suggests that countries with more ambitious renewable electricity targets would have higher net regional impacts than countries with less ambitious targets, but existing regional inequalities are further reduced with any targets.

With this study, we extend the current scientific literature in four aspects. First, we provide a more holistic picture of the regional impacts associated with the electricity system transition by quantifying employment impacts, greenhouse gas and particulate matter emissions, and land use at high NUTS-3 spatial detail. Second, in contrast to existing modeling studies on regional equality, this study accounts for the largest region of six European countries with 650 spatial NUTS-3 units and takes into account the technical feasibility in terms of hourly operation, transmission, and storage. Third, we propose and quantify new dimensions of regional equality, namely equality of system costs, employment, greenhouse gas and particulate matter emissions, and land use. Last, our modeling approach presents the most extensive application of MGA to date for spatial electricity sector modeling.

The results of this study are partly affected by four key assumptions. First, the role of policies, like subsidies or taxes, in shaping the technology choice are covered only implicitly by modeling 100 cost-optimal and near-optimal scenarios. The change in regional distribution of costs and benefits due to policies is also not quantified, and therefore future research could further explore the role of policies. Second, we ensure that each country meets its annual electricity generation from indigenous generation plants, which is consistent with supply security consideration in individual countries, but makes the scenarios reflect international cooperation to a lesser extent (see “Methods”). This constraint limits the lower regional equality values of the model, but it does not change our finding that cost efficiency encourages regional inequalities, while the goals of regional equality of system costs or maximum renewable electricity generation drive regional equalities. Third, we account for the electricity sector alone, instead of the entire energy system, and future studies could explore the regional impacts with whole system models. Fourth, we have chosen the short- to medium-term horizon of 2035 for the analysis as this is the timeframe of national electricity targets, whereas future work could explore transition to mid-century. Nevertheless, this study demonstrates how MGA methodology can be applied to large-scale spatially explicit electricity system models to quantify the regional impacts of the electricity sector transition under consideration of multiple societal aims, other than costs. These types of studies and methodologies thus can foster a more holistic discussion on the regional implications of the electricity sector transition by linking national energy and climate targets with impacts on local communities.

## Methods

### Modeling approach

**Fig. 8:**
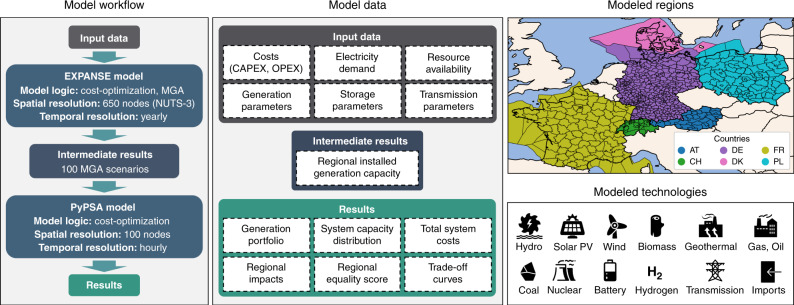
Overview of model workflow, data, regions and technologies. MGA modeling to generate alternatives; NUTS-3 third-level nomenclature of territorial units for statistics.

Our modeling approach (Fig. 8) soft links two optimization models called EXPANSE^27,31^ and PyPSA^32^ to assess feasible spatial distributions of electricity system infrastructure for Central Europe in 2035. Initially, we run the EXPANSE model, which applies MGA method^27,31,33^ to compute 100 maximally different scenarios of electricity generation capacities and locations. Next, we run each of these generation scenarios with the PyPSA model to optimize hourly electricity generation and long-term investment in storage and transmission capacity. Such soft linking of models allows to keep the computational time reasonable, and is also meaningful because the deployment of electricity generation can be expected to deviate significantly from cost optimality^27,38^, whereas transmission infrastructures are planned in a more centralized way and power plants are operated following the cost-driven market principles. From these 100 MGA scenarios, we estimate the associated regional impacts regarding system costs, employment, greenhouse gas emissions, particulate matter, and land use. We analyze these regional impacts for four distinct scenarios with frozen generation capacity, minimum system costs, maximum regional equality of system costs, and maximum renewable electricity generation. We evaluate how even these impacts are distributed across regions by applying the Gini index^37^. All input data and results have a high spatial resolution of NUTS-3^30^ and are available on Zenodo^36^.

### Soft-linked EXANSE-PyPSA model

From the two models that we link, EXPANSE^27,31^ is a spatially explicit, bottom-up, technology-rich, single-year electricity system model for 2035 with annual resolution and high spatial resolution. The model has 650 nodes representing all individual NUTS-3 regions of Central Europe. Its unique feature is that it applies the MGA method^27,33,34^ to compute many spatial allocation scenarios of electricity generation capacity with cost-optimal and near-optimal costs. The principle of MGA is to relax the cost-optimal spatial allocation with an acceptable relative cost increase called slack and to search for maximally different scenarios. For each scenario, we allow EXPANSE to randomly vary the slack between 0 and 20% above cost-optimal total electricity generation costs of all six countries to compute 100 near-optimal scenarios. The maximum slack of 20% has been selected based on the cost deviations of 9–23% found in a modeling study^38^, which compared the cost-optimal and actual UK electricity system transition between 1990 and 2014. The slack is applied to the total electricity generation costs of all six countries, whereas each of the 100 MGA runs leads to various slack outcomes that are sometimes more and sometimes less even among the countries.

The second model PyPSA^32,39^ is an electricity system model without MGA and with lower spatial resolution. While the purpose of EXPANSE is to generate large numbers of spatial allocation scenarios of generation capacity, the purpose of PyPSA is to complement EXPANSE post hoc with hourly computations of electricity generation and assessment of storage and transmission requirements. The PyPSA model has 100 nodes representing a simplified grid of our study region. The objective of PyPSA is to minimize the total annualized system costs of each scenario set by EXPANSE. We soft link the EXPANSE and PyPSA models as follows: first, EXPANSE allocates the electricity generation capacities within NUTS-3 regions. Next, we aggregate these regional capacities to the closest grid node within the same country. Finally, PyPSA optimizes for each grid node the hourly operation of electricity generation, storage, and transmission, and the annualized investment in storage and transmission capacity. Due to the computational intensity of computing many scenarios with PyPSA, we simplify the actual grid layout with k-means clustering^32^ and apply a rolling horizon optimization technique with historic hydro reservoir levels^40^ as set points.

### Input data and assumptions

The model includes a broad portfolio of renewable and conventional electricity generation, storage, and transmission technologies. Renewable electricity generation includes wind (onshore and offshore), solar PV (open-field and rooftop), hydropower (large dams, large run-of river and small hydropower), biomass (biogas, woody biomass, and waste), and geothermal. Conventional electricity generation includes nuclear, hard coal, lignite, gas, and oil. Storage includes battery, hydrogen, and pumped hydropower storage. Transmission includes high voltage alternating current (HVAC) and high voltage direct current (HVDC) transmission lines.

As a starting point, we estimate the electricity demand at an annual resolution for each NUTS-3 region (used by EXPANSE) and at an hourly resolution for each grid node (used by PyPSA). We collect the assumed annual demand in 2035 for each country from the EU Reference Scenario^41^ and the Swiss Energy Strategy^42^. These scenarios foresee that the total electricity demand in our study region will increase by 8% until 2035. For each NUTS-3 region, we spatially disaggregate this demand with spatial weights from a modeling study^43^. They are based on the regional distribution of population and electricity-intensive industries. For each grid node, we spatially and temporally disaggregate the electricity demand with spatial and temporal weights. We derive these weights from an adapted version of the European PyPSA model^44^ with our specific 100 node grid layout. They are based on regional population and gross domestic product, and hourly electricity demand^45^.

We model the renewable generation potentials and current generation capacities. For wind and solar PV, we collect spatially explicit generation potentials from a modeling study^43^. We limit these potentials with maximum growth rates assumed until 2035, which we derive from the ambitious European 1.5 Tech scenario^46^ and the Swiss Energy Strategy 2050^42^. We determine the hourly availability of these generation potentials by using the Renewables Ninja tool^47,48^. For hydropower, we collect country-specific generation potentials from the PRIMES model^49^. We model the hourly availability of these potentials and hourly reservoir levels with the tools included in the PyPSA model^32^. For biomass, we collect spatially explicit generation potentials from the JRC ENSPRESO biomass study^50^ (Supplementary Table 1). For geothermal, we estimate the spatially explicit generation potentials based on the temperatures at 5000 m depth^51^ (Supplementary Table 2). We estimate current generation capacities by merging the open power systems dataset^52^, the JRC hydropower dataset^53^, and national electricity statistics^54^. To amend the lack of spatial representation in some countries, we add a database of solar PV panels in Switzerland^23^, wind turbine locations in Austria^55^, and regional biomass, solar PV, and wind capacities in Austria^56^.

For nuclear, we estimate the spatially explicit generation potentials by assuming that existing nuclear power plants can be safely operated until the end of their lifetimes and that no additional power plants can be built. One exception is an optional new 1.5 GW nuclear power plant near Poland’s Baltic coast. For hard coal and lignite, we follow the same approach as for nuclear but do not include any possibilities for new power plants. For gas, we estimate the spatially explicit generation potentials based on the highest gas electricity generation found in representative modeling studies for Austria^57^, Denmark^58^, France^59^, Germany^60^, Poland^61^, and Switzerland^42^. We spatially disaggregate these generation potentials to NUTS-3 regions where currently hard coal, lignite, and gas power plants exist, and for gas in Switzerland, to highly populated NUTS-3 regions with more than 300,000 inhabitants. For oil, we follow the same approach as for hard coal and lignite. We assess current generation capacities by merging the open power systems dataset^62^ and the national electricity statistics^54^.

For battery and hydrogen storage, we follow a greenfield approach with initially zero storage capacities in all regions. PyPSA can extend these capacities, depending on the storage requirements of each scenario. For pumped hydropower storage, we assume that existing capacities will not change until 2035. For both HVDC and HVAC transmission lines, we collect the transmission line capacities from the European PyPSA dataset^44^, which we adapt to our 100 node grid layout. This dataset accounts for the currently existing transmissions lines, which it extracts from the GridKit dataset^63^. Additionally, it includes the HVDC projects planned by the TYNDP 2018^64^ that are at least in the permitting phase. These transmission capacities can be extended by PyPSA to ensure grid security for each MGA scenario. To model grid security, we assume that hourly power flows over each transmission line cannot be above 70% of its thermal limit. If that limit is reached, PyPSA extends the transmission line capacity.

The EXPANSE model considers levelized electricity generation costs for the year 2035 to optimize regional electricity generation capacity, which we calculate with assumed techno-economic parameters (Supplementary Table 3). The PyPSA model considers annualized capital and variable costs to optimize hourly electricity generation, storage, and transmission capacity, which we calculate with techno-economic parameters for electricity generation (Supplementary Table 3), storage (see Supplementary Table 4), and transmission (Supplementary Table 5). We assume a 5% weighted average cost of capital and do not include any subsidies and taxes (neither feed-in tariffs nor carbon tax). We define the sum of annualized capital and variable costs from electricity generation, storage, and transmission as total system costs.

### Definitions of distinct scenarios

We analyze all 100 MGA scenarios and select four distinct scenarios for an in-depth look (Table 1). The first scenario with minimum system costs is the MGA scenario with the least total system costs. The second scenario with maximum regional equality is the MGA scenario with the most regionally even distribution of system costs. The third scenario with maximum renewable electricity generation is the MGA scenario with the highest annual renewable electricity generation. The fourth scenario with frozen generation capacity is not an MGA scenario, and it assumes the same electricity generation capacity for the year 2035 as in 2018. This scenario is used to measure the impacts of various scenarios as compared to today’s generation mix in a harmonized way with the same costs and other assumptions as in 2035. This setup equals to a total of 101 spatially explicit scenarios that we assess.

**Table 1:** Definition of scenarios.

Scenario	Electricity generation capacity	Storage and transmission capacity	Electricity demand	Country-level targets for 2035 (modeled as constraints)
Frozen generation capacity	Exogenously defined as in the year 2018 (424 GW)	Optimized by the model	Exogenously defined (1615 TWh year^−1^)	No targets
100 MGA scenarios (including the scenarios of minimum system costs, maximum regional equality of system costs, and maximum renewable electricity generation)	Optimized by the model	Optimized by the model	Exogenously defined (1615 TWh year^−1^)	Austria: 100% renewable electricity generation^66^ Denmark: 100% renewable electricity generation^67^ France: <50% nuclear electricity generation^68^ Germany: >70% renewable electricity generation^69^ Poland: <40% hard coal and lignite electricity generation^61^ Switzerland: >11.4 TWh year^−1^ renewable electricity generation without hydropower^70^

Electricity generation capacity for the scenario with frozen generation capacity is exogenously defined and kept constant in the EXPANSE model (Table 1). In contrast, electricity generation capacities of all 100 MGA scenarios are optimized by the EXPANSE model. Storage and transmission capacities are optimized by the PyPSA model for the scenario with frozen generation capacity and also for all 100 MGA scenarios. Electricity demand is exogenously defined for the scenario with frozen generation capacity and all 100 MGA scenarios, with an overall electricity demand increase of 8% predicted until 2035 for our study region. The model ensures that all scenarios have sufficient generation capacities to cover this demand increase by running generation capacities at higher capacity factors and by extending transmission and storage capacities. In addition, the modeled six countries are not isolated and can import electricity from abroad. Country-level electricity generation targets are defined for all 100 MGA scenarios, but not the scenario with frozen generation capacity. We derive these values from the national electricity targets of each modeled country (shown in Table 1). Finally, we add a constraint to the EXPANSE model (see the model formulation in the Supplementary methods and the constraint definitions in the Supplementary Table 6) that each country meets its domestic annual electricity demand from indigenous electricity generation plants. Hourly electricity import and export through transmission lines is included by the PyPSA model and does not add to the annual electricity demand. This constraint ensures that each country has own indigenous generation and does not outsource large portions of generation abroad, which would be unlikely due to national supply security concerns. This is a reasonable assumption for the modeled six countries, because on annual basis these countries currently cover most of their annual electricity demand with indigenous generation and yet still trade with low net electricity imports.

### Evaluating regional impacts and regional equality

We assess the regional technical, economic, social, climate change, health, and environmental impacts as defined in Table 2. Technical and economic impacts are direct results of the model. All other regional impacts are estimated with impact factors (e.g., direct CO_2_ emissions per MWh of coal electricity or direct jobs per MW of wind capacity) by using data from previous peer-reviewed studies (Supplementary Table 7). For employment, we calculate the regional impact by multiplying the regional electricity generation and storage capacity (in MW) with technology-specific impact factors. For greenhouse gas emissions, particulate matter, and land use, we calculate the regional impact by multiplying the regional technology-specific electricity generation (in MWh) with associated impact factors.

**Table 2:** Definition of regional impacts and units.

Impact category	Regional impact	Definition	Unit
Technical impact	Electricity system infrastructure	Electricity generation, storage, and transmission capacity	MW
Economic impact	Total system costs	Annual capital and variable costs (i.e., fuel, operation, and maintenance) for electricity generation, storage, and transmission	EUR year^−1^
Social impact	Employment	Annual direct employment for electricity generation and storage. For all technologies, we include jobs in construction, installation, operation, maintenance, and decommissioning. For biomass, coal, and lignite, we additionally include jobs in fuel extraction and transport.	Jobs year^−1^
Climate change impact	Greenhouse gas emissions	Annual direct greenhouse gas emissions from fuel combustion for electricity generation	Tons CO_2-eq_ year^−1^
Health impact	Particulate matter	Annual direct particulate matter formation from fuel combustion for electricity generation	Tons PM_10-eq_ year^−1^
Environmental impact	Land use	Direct land use for electricity generation	km^2^

After modeling the regional impacts, we determine how even these are distributed. Specifically, we evaluate the evenness of regional system costs per capita, direct employment for electricity generation and storage, direct greenhouse emissions and particulate matter per capita, and direct land use per total area to generate electricity. For each impact, a scenario is defined as least regionally equal if the impact (e.g., system costs) is located in only one region. In contrast, a scenario is most regionally equal if all regions have the same values for that specific impact. As proposed in previous studies^26,27^, we measure regional equality with the Gini index^37^. We define a more intuitive measure for regional equality, with 0% being the lowest and 100% being the highest regional equality score, by adapting the Gini index formulation^65^ with Eq. (1):

1 $${\mathrm{Regional}}\;{\mathrm{equality}} = {\mathrm{1}} - {\mathrm{Gini}}\;{\mathrm{index}} = 1 - \frac{{\mathop {\sum}\nolimits_{i = 1}^n {\mathop {\sum}\nolimits_{j = 1}^n {| {x_i - x_j} |} } }}{{2n^2\bar x}},$$

where *x* is the examined regional impact (e.g., system costs or jobs per capita) in each NUTS-3 region, *n* is the total number of NUTS-3 regions, and indices *i* and *j* represent the NUTS-3 region nodes. We apply Eq. (1) to assess regional equality of system costs, employment, greenhouse gas emissions, particulate matter, and land use. Note that our scenario definitions do not change, and maximum regional equality remains the scenario with the most regionally even distribution of system costs (and not of other impacts).

## Supplementary information

Supplementary Information

Supplementary Data 1

## Data Availability

The data on costs, potentials, capacity factors, demand, and regional impacts that support the findings of this study are available in Zenodo with the identifier 10.5281/zenodo.3967297. Source data are provided with this paper.

## Code availability

The code used for analysis in this study is available from the corresponding author upon request.
