# Supplementary material for: Regional impacts of electricity system transition in Central Europe until 2035
Source: Nat Commun. 2020 Oct 2;11:4972. doi: 10.1038/s41467-020-18812-y (PMC7532153; doi:10.1038/s41467-020-18812-y)
Supplement: Supplementary file 1 — Supplementary Information [file 41467_2020_18812_MOESM1_ESM.pdf]

Supplementary Information for Regional impacts of electricity system transition in Central Europe until 2035 by Sasse et al.

Supplementary Table 1: Fuels used by each biomass technology type and cost ranges.

Technology type	Technology name	Fuel commodity	Sector	Feedstock cost range (EUR GJ^{-1}) [1]
Biogas	Anaerobic digestion	Manure	Agriculture	3.2-7.6
		Sewage sludge	Waste	6
Woody biomass	Grate furnace steam turbine	Grassy crops	Agriculture	2.8-8.5
		Willow		7-14.4
		Agricultural residues		2.3-5.6
		Poplar		9.5-19.3
		Additionally harvestable stemwood	Forestry	2.6-9.4
		Logging residues		1.4-6.5
		Landscape care		2.2-3.3
		Woodchips & pellets		1.4-3.2
		Sawdust		1.3-2.3
Waste	Municipal solid waste incinerator	Municipal waste	Waste	0 (assumption)

Note: Feedstock costs are spatially-explicit and vary between the indicated minimum and maximum values.

Supplementary Table 2: Maximum installable capacity of geothermal plants.

Temperature range	Maximum installable capacity
< 140°C	0 MW
140 - 160°C	2.8 MW
160 - 200°C	5.5 MW
200 - 240°C	14.6 MW

Note: Assumed maximum installable capacities of enhanced geothermal systems depend on rock temperature at 5'000m depth for each NUTS-3 region.

Supplementary Table 3: Techno-economic parameters of electricity generation in 2035.

**Technology**	**CAPEX (EUR kW^{-1})**	**FOM (% of CAPEX $year^{-1}$)**	**VOM (EUR MWh_{el}^{-1})**	**Efficiency (%)**	**Fuel (EUR MWh_{el}^{-1})**	**Lifetime (years)**	**Capacity factor (-)**	**LCOE (EUR MWh_{el}^{-1})**
Renewable electricity generation								
Wind (onshore)	1584 [3]	1.71 [4]	1.3 [5]	-	-	25 [4]	0.06-0.51	32-254
Wind (offshore)	2322 [3]	2.5 [4]	2.6 [5]	-	-	25 [4]	0.41-0.47	60-72
Solar PV (open field)	465 [4]	2.5 [4]	0 [6]	16% [6]	-	25 [4]	0.12-0.18	27-42
Solar PV (rooftop)	810 [4]	2.5 [4]	0 [6]	16% [6]	-	25 [4]	0.10-0.15	58-88
Large hydro dams	2200 [6]	1 [6]	3 [6]	90% [6]	-	60 [6]	0.23	71
Large run of river	3370 [6]	1.5 [6]	3 [6]	90% [6]	-	60 [6]	0.49	56
Small hydro	5620 [6]	1.5 [6]	3 [6]	90% [6]	-	60 [6]	0.47	95
Biogas	2640 [6]	4.1 [6]	3.1 [6]	46% [7]	29-53 [1]	20 [6]	0.6	93-117
Woody biomass	2260 [6]	2.2 [6]	3.5 [6]	46% [7]	19-46 [1]	25 [6]	0.6	63-90
Waste	5055 [6]	3 [6]	6.9 [6]	46% [7]	0-2 [1]	25 [6]	0.7	90-92
Geothermal	9000 [6]	1.9 [7]	0 [6]	24% [6]	-	40 [8]	0.76	105-626
Conventional electricity generation								
Nuclear	4500 [3]	1.9 [4]	9 [9]	33.7% [6]	10 [9]	60 [8]	0.82	64
Hard coal	1800 [3]	2.5 [4]	3.6 [6]	46.4% [6]	40 [9]	40 [8]	0.47	80
Lignite	1800 [3]	2.5 [4]	4.5 [6]	44.7% [6]	25 [9]	40 [8]	0.73	53
Gas	900 [3]	2.5 [4]	2 [6]	50.0% [6]	75 [9]	30 [8]	0.32	106
Oil	400 [7]	1.5 [4]	3 [7]	39.4% [6]	130 [9]	30 [8]	0.15	157

Abbreviations: CAPEX = capital expenditures, FOM = fixed operation costs, VOM = variable operation costs, LCOE = levelized cost of electricity generation. Capacity factors and LCOE are spatially explicit and vary between the indicated minimum and maximum values.

Supplementary Table 4: Techno-economic parameters of storage in 2035.

Technology	CAPEX (EUR kW^{-1})	FOM (% of CAPEX $year^{-1}$)	VOM (EUR MWh_{el}^{-1})	Efficiency (%)	Lifetime (years)	Extendable capacity
Pumped hydro storage	2200 [6]	1 [6]	3 [6]	75% [6]	60 [6]	No
Battery storage	1373 [10]	1 [10]	-	84% [10]	13 [10]	Yes
Hydrogen storage	4605 [10]	1 [10]	-	40% [10]	18 [10]	Yes

Abbreviations: CAPEX = capital expenditures, FOM = fixed operation costs, VOM = variable operation costs.

Supplementary Table 5: Techno-economic parameters of transmission lines in 2035.

Component	CAPEX of lines (EUR $MWkm^{-1}$)	CAPEX of inverters (EUR MW^{-1})	FOM (% of CAPEX $year^{-1}$)	Lifetime (years)
HVAC<br>- overhead line	400 [11]	-	2 [11]	40 [11]
HVDC<br>- overhead line	400 [11]	-	2 [11]	40 [11]
HVDC<br>- submarine line	2000 [11]	-	2 [11]	40 [11]
HVDC<br>- inverter pair	-	150000 [11]	2 [11]	40 [11]

Abbreviations: HVAC = high voltage alternating current transmission line, HVDC = high voltage direct transmission line, CAPEX = capital expenditures, FOM = fixed operation costs.

Supplementary Table 6: Country-level targets and modeled constraints for 2035.

Country	Defined target	Modelled constraint	Technologies in S	δ_N
Austria	100% renewable electricity generation[12]	$E_{\text{target};N,S} =$ 81.67 TWh	Biogas, biomass waste, woody biomass, geothermal, large hydro dams, large run-of-river, small hydro, onshore wind, solar rooftop PV, solar open-field PV	1
Denmark	100% renewable electricity generation[13]	$E_{\text{target};N,S} =$ 36.43 TWh	Biogas, biomass waste, woody biomass, geothermal, large hydro dams, large run-of-river, small hydro, onshore wind, offshore wind, solar rooftop PV, solar open-field PV	1
France	< 50% nuclear electricity generation[14]	$E_{\text{target};N,S} =$ 301.96 TWh	Nuclear	-1
Germany	> 70% renewable electricity generation[15]	$E_{\text{target};N,S} =$ 428.14 TWh	Biogas, biomass waste, woody biomass, geothermal, large hydro dams, large run-of-river, small hydro, onshore wind, offshore wind, solar rooftop PV, solar open-field PV	1
Poland	< 40% hard coal and lignite electricity generation[16]	$E_{\text{target};N,S} =$ 84.62 TWh	Hard coal, lignite	-1
Switzerland	> 11.4 TWh year^{-1} renewable electricity generation without hydropower[17]	$E_{\text{target};N,S} =$ 11.4 TWh	Biogas, biomass waste, woody biomass, geothermal, onshore wind, solar rooftop PV, solar open-field PV	1

Note: see Supplementary Methods for the implementation of the country-level targets and constraints in the EXPANSE model.

Supplementary Table 7: Impact factors for electricity generation and storage.

Technology	Regional direct employment (Jobs MW^{-1})	Direct greenhouse gas emissions ($tCO_{2\text{-eq}}$ MWh_{el}^{-1})	Direct particulate matter formation ($gPM_{10\text{-eq}}$ MWh_{el}^{-1})	Direct land-use (m^2 MWh_{el}^{-1})
Renewable electricity generation				
Wind (onshore)	0.395 [18]	-	-	2.280 [19]
Wind (offshore)	1.343 [18]	-	-	-
Solar PV (open field)	0.336 [18]	-	-	0.700 [19]
Solar PV (rooftop)	0.336 [18]	-	-	-
Large hydro dams	0.857 [18]	-	-	4.100 [19]
Large run of river	1.326 [18]	-	-	0.003 [19]
Small hydro	1.326 [18]	-	-	0.003 (assumption)
Biogas	1.300 [18]	0.31 [9]	200.00 [20]	12.650 [19]
Woody biomass	1.300 [18]	0.07 [9]	334.00 [20]	12.650 [19]
Waste	1.163 [18]	0.07 [9]	100.00 [20]	0.310 (assumption)
Geothermal	0.667 [18]	0.06 [9]	1.00 [20]	0.740 [19]
Conventional electricity generation				
Nuclear	0.818 [18]	-	-	0.120 [21]
Hard coal	0.694 [18]	0.75 [9]	206.00 [20]	0.390 [19]
Lignite	1.552 [18]	0.82 [9]	320.00 [20]	0.900 [19]
Gas	0.296 [18]	0.34 [9]	101.00 [20]	0.310 [19]
Oil	0.296 [18]	0.67 [9]	200.00 [20]	0.310 (assumption)
Storage				
Pumped hydro	0.857 [18]	-	-	-
Battery	0.384 [22]	-	-	-
Hydrogen	0.269 [22]	-	-	-

Note: Employment impact includes annual direct employment for electricity generation and storage, including jobs in construction, installation, operation, maintenance, and decommissioning. For biomass, coal, and lignite, we additionally include jobs in fuel extraction and transport. Greenhouse gas emissions impact includes annual direct greenhouse gas emissions from fuel combustion for electricity generation. Particulate matter impact includes annual direct particulate matter emissions from fuel combustion for electricity generation. Land use impact includes direct land use for electricity generation.

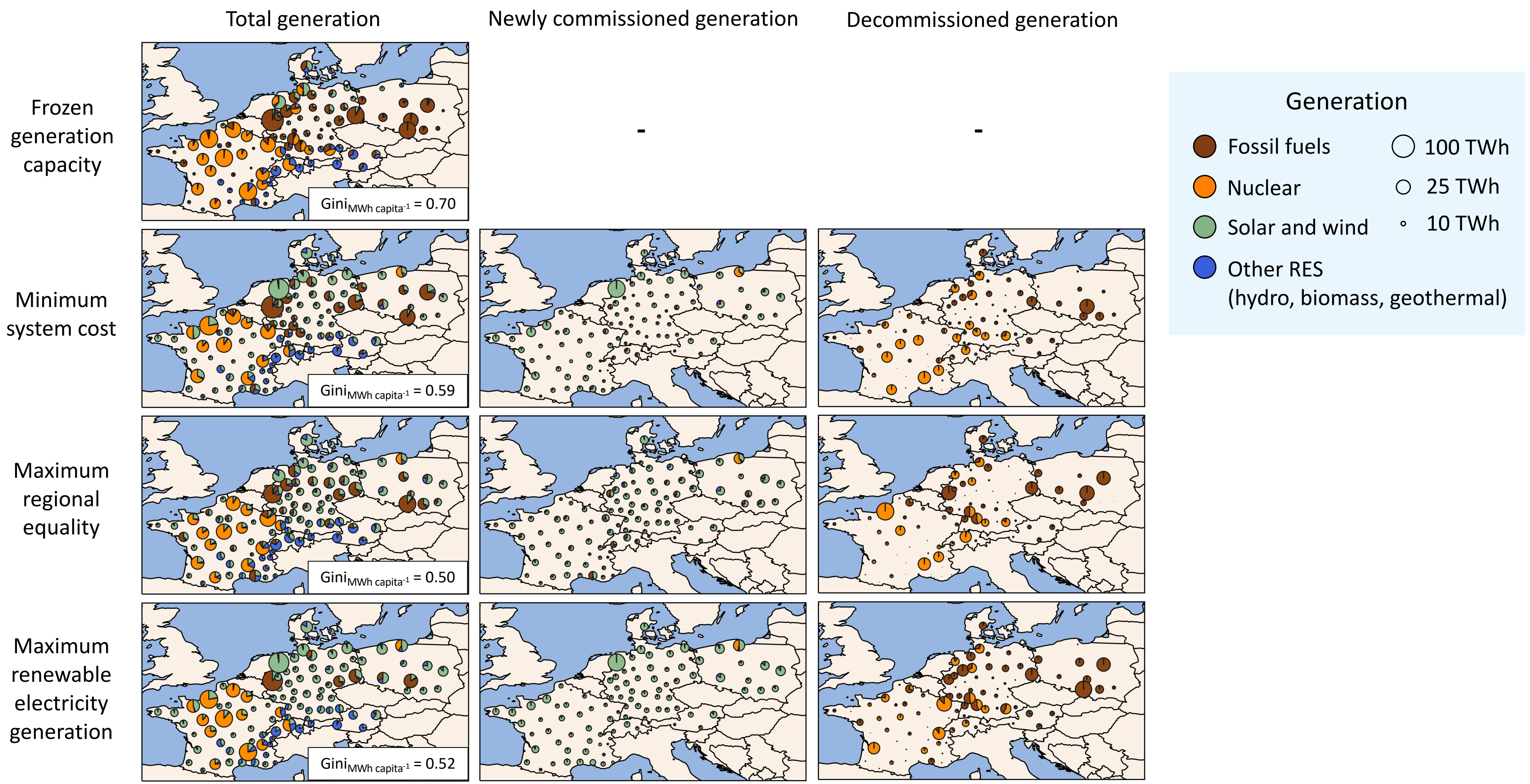

Supplementary Fig. 1: Regional changes in electricity generation in 2035. Total generation, newly commissioned generation and decommissioned generation as compared to 2018 at each of the 100 grid nodes for the four distinct scenarios. Values shown in TWh year^{-1} for the year 2035. In the scenarios of minimum system cost, maximum regional equality, and maximum renewable electricity generation, each country meets its electricity targets for 2035 (see Methods).

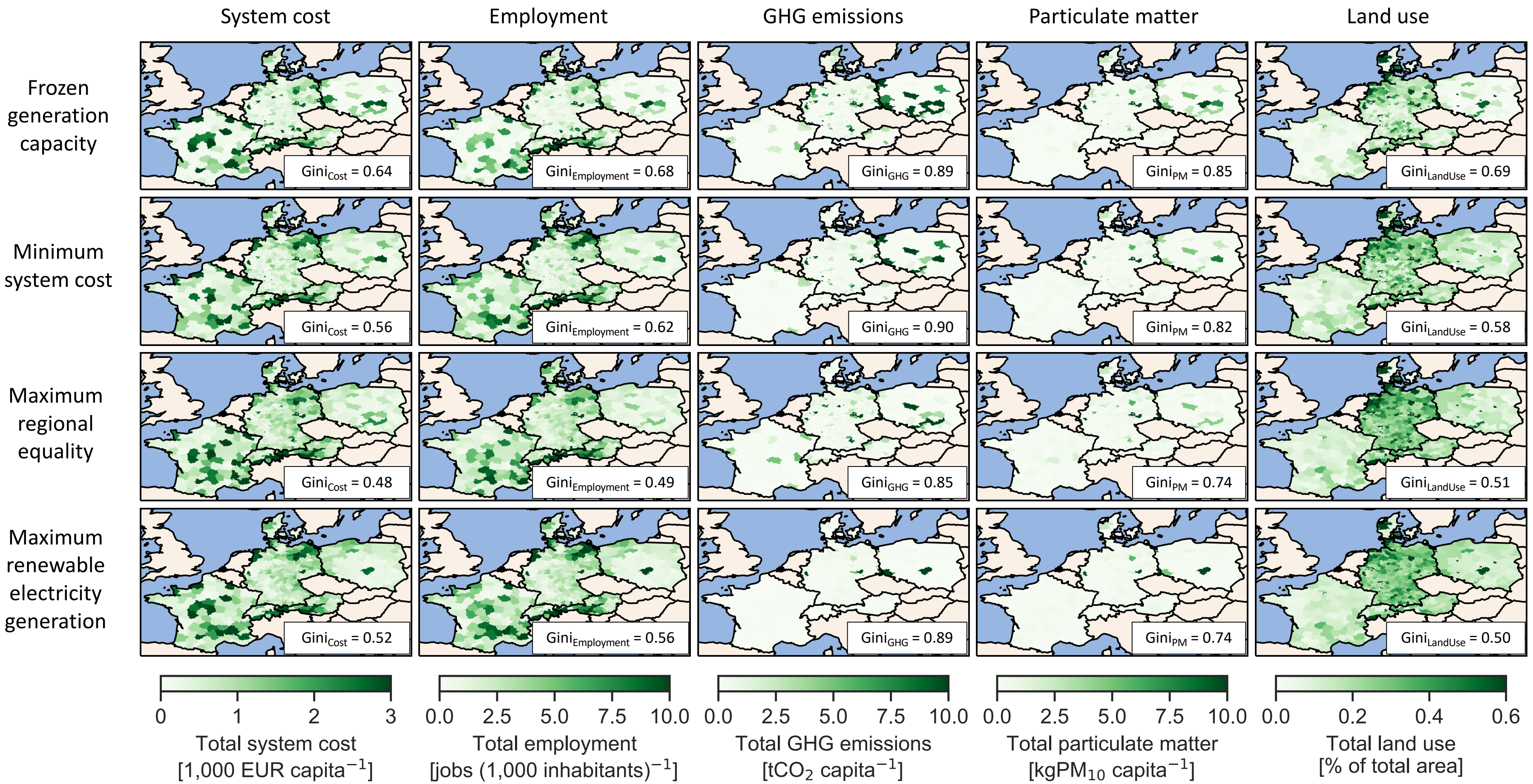

Supplementary Fig. 2: Regional impacts from generation, storage, and transmission in 2035. Values show total regional system costs, employment, greenhouse gas (GHG) emissions, particulate matter emissions, and land use for the four distinct scenarios. A Gini index of 1.0 indicates perfect regional inequality and a Gini index of 0.0 indicates perfect regional equality. In the scenarios of minimum system cost, maximum regional equality, and maximum renewable electricity generation, each country meets its electricity targets for 2035 (see Methods). Source data are provided in Supplementary Data 1.

Supplementary Methods

EXPANSE model

Nomenclature:

S	electricity generation technology group label;
s	electricity generation technology label;
N	country label;
n	NUTS-3 region label;
D_N	annual electricity generation to cover demand in each country N, in MWh year^{-1};
$x_{n,s}$	generated electricity by technology s in NUTS-3 region n, in MWh year^{-1};
$x_{\max;n,s}$	maximum annual resource potential by technology s in NUTS-3 region n, in MWh year^{-1};
$x_{\min;n,s}$	minimum annual resource potential by technology s in NUTS-3 region n, in MWh year^{-1};
$E_{\text{target};N,S}$	technology-specific electricity generation target for all technologies within each technology group S in each country N, in MWh year^{-1};
δ_N	binary variable for indicating if the electricity generation target $E_{\text{target};N,S}$ of each country is a minimum or a maximum electricity generation target, where $\delta_N = \begin{cases} 1 & \text{if } E_{\text{target};N,S} \text{ is a minimum target} \\ -1 & \text{if } E_{\text{target};N,S} \text{ is a maximum target} \end{cases}$
$\alpha_{n,s}$	randomly drawn number from a uniformly distributed set {-1,0,1} for each NUTS-3 region n and generation technology s;
$c_{n,s}$	levelized cost of electricity (including investment, operation and maintenance, and fuel costs) for each NUTS-3 region n and generation technology s, in EUR MWh^{-1};
C_{slack}	the cost constraint (moving slack[23]), which varies between 0% and 20% above minimum total levelized costs, in EUR MWh^{-1};
$C_{\min}$	minimum total levelized costs, in EUR MWh^{-1}.

EXPANSE[24,25] is a linear programming model that generates a diverse set of technically-feasible and cost-effective electricity supply scenarios that meet economic, environmental, and technical constraints. This is achieved by applying the Modeling to Generate Alternatives

(MGA) method[24,26,27]. The principle of MGA is to relax the cost-optimal solution and use a modified problem formulation to search the near-optimal solution space for a wanted number of scenarios that are maximally different, but still within pre-defined cost limits. For each MGA scenario, this cost limit (called moving slack[23]) is randomly drawn from a uniform distribution and bounded between 0% and 20% above cost-optimal costs. The MGA scenarios are generated by applying the following steps:

Step 1: Solve the original problem to obtain the cost-optimal solution and its costs:

$$C_{\min} = \min_{x} \sum_{n,s} c_{n,s} x_{n,s} \tag{1}$$

subject to

$$x_{\min;n,s} \leq x_{n,s} \leq x_{\max;n,s} \qquad \forall n, s \tag{2}$$

$$\sum_{n \in N, s} x_{n,s} = D_N \qquad \forall N \tag{3}$$

$$\begin{cases} \sum_{n \in N, s \in S} x_{n,s} \geq E_{\text{target};N,S}, \ \text{if } \delta_N = 1 & \forall N, S \\ \sum_{n \in N, s \in S} x_{n,s} \leq E_{\text{target};N,S}, \ \text{if } \delta_N = -1 & \forall N, S \end{cases} \tag{4}$$

Equations (2), (3), and (4) guarantee that each MGA scenario is technically feasible, meets the annual demand constraints, and meets the national technology-specific electricity generation target. Each country-level target is modelled as a constraint as shown in Supplementary Table 6.

Step 2: For each MGA scenario, obtain the randomly drawn moving slack from a uniform distribution, where:

$$C_{\min} \leq C_{\text{slack}} \leq 1.2 \cdot C_{\min} \tag{5}$$

Step 3: For each MGA scenario, add the moving slack as an additional cost constraint to the original problem formulation (1)-(4):

$$\sum_{n,s} c_{n,s} x_{n,s} \leq C_{\text{slack}} \tag{6}$$

Step 4: For each MGA scenario, remove the previous objective function (1) and instead optimize the following objective function:

$$\max_x \sum_{n,s} \alpha_{n,s} x_{n,s} \quad (7)$$

The objective function (7) is subject to constraints, defined by Equations (2), (3), (4), and (6).

Step 5: Iterate steps 2 - 4 until a wanted number, in our case 100, of MGA scenarios is found.

PyPSA model

The code and documentation of the PyPSA[28,29] model can be found on GitHub: https://github.com/PyPSA/PyPSA

The PyPSA model is set up for this study to include 100 grid nodes. We exclude the electricity generation capacity optimization from the objective function, as these are pre-defined by the EXPANSE model. We then soft-link the EXPANSE and PyPSA models as follows: first, EXPANSE allocates the electricity generation capacities within NUTS-3 regions. Next, we aggregate these regional capacities to the closest grid node within the same country. Finally, PyPSA optimizes for each grid node the hourly operation of electricity generation, storage, and transmission, and the annualized investment in storage and transmission capacity.

Supplementary References

1. Ruiz, P. *et al.* ENSPRESO - an open, EU-28 wide, transparent and coherent database of wind, solar and biomass energy potentials. *Energy Strateg. Rev.* **26**, 100379 (2019).
2. EP. *Regulation (EC) No 1059/2003 of the European Parliament and of the Council of 26 May 2003 on the Establishment of a Common Classification of Territorial Units for Statistics (NUTS)*. (European Parliament (EP), Brussels, 2019).
3. IEA. *World Energy Model - Documentation (2019 Version)*. (International Energy Agency (IEA), Paris, 2019).
4. Fraunhofer ISE. *Stromgestehungskosten Erneuerbare Energien*. (Fraunhofer Institute for Solar Energy Systems (ISE), Freiburg, 2018).
5. *Technology Data for Energy Plants Generation of Electricity and District Heating*. (Danish Energy Agency, Copenhagen, 2019).
6. JRC. *ETRI 2014 - Energy Technology Reference Indicator projections for 2010-2050*. (Joint Research Centre (JRC), Petten, 2014).
7. Schröder, A., Kunz, F., Meiss, J., Mendelevitch, R. & von Hirschhausen, C. *Current and Prospective Costs of Electricity Generation until 2050*. (German Institute for Economic Research (DIW), Berlin, 2013).
8. IEA. *Projected Costs of Generating Electricity*. (International Energy Agency (IEA), Paris, 2015).
9. Bauer, C. *et al. Potentials, costs and environmental assessment of electricity generation technologies*. (Swiss Federal Office of Energy (SFOE), Bern, 2017).
10. Schmidt, O., Melchior, S., Hawkes, A. & Staffell, I. Projecting the Future Levelized Cost of Electricity Storage Technologies. *Joule* **3**, 81–100 (2019).
11. Hörsch, J., Hofmann, F., Schlachtberger, D. & Brown, T. PyPSA-Eur: An open optimisation model of the European transmission system. *Energy Strateg. Rev.* **22**, 207–215 (2018).
12. *Integrated National Energy and Climate Plan for Austria*. (Republic of Austria - Federal Ministry for Sustainability and Tourism, Vienna, 2019).
13. *Denmark's Integrated National Energy and Climate Plan*. (Danish Ministry of Climate Energy and Utilities, Copenhagen, 2019).
14. *Plan National Integre Energie-Climat de la France*. (Ministère de la Transition écologique, Paris, 2020).
15. *Koalitionsvertrag 2018 - Ein neuer Aufbruch für Europa, Eine neue Dynamik für*

Deutschland, Ein neuer Zusammenhalt für unser Land. (Presse- und Informationsamt der Bundesregierung, Berlin, 2018).

16. *Executive Summary of Poland's National Energy and Climate Plan for the Years 2021-2030 (NECP PL)*. (Polish Ministry of State Assets, Warsaw, 2020).
17. SFC. *Energiegesetz (EnG)*. (Swiss Federal Council (SFC), Bern, 2016).
18. Kis, Z., Pandya, N. & Koppelaar, R. Electricity generation technologies: Comparison of materials use, energy return on investment, jobs creation and CO2 emissions reduction. *Energy Policy* **120**, 144–157 (2018).
19. Fthenakis, V. & Kim, H. C. Land use and electricity generation: A life-cycle analysis. *Renew. Sustain. Energy Rev.* **13**, 1465–1474 (2009).
20. Luderer, G. *et al.* Environmental co-benefits and adverse side-effects of alternative power sector decarbonization strategies. *Nat. Commun.* **10**, 1–13 (2019).
21. Maxim, A. Sustainability assessment of electricity generation technologies using weighted multi-criteria decision analysis. *Energy Policy* **65**, 284–297 (2014).
22. Ram, M., Aghahosseini, A. & Breyer, C. Job creation during the global energy transition towards 100% renewable power system by 2050. *Technol. Forecast. Soc. Change* **151**, 119682 (2020).
23. Li, F. G. N. & Trutnevyte, E. Investment appraisal of cost-optimal and near-optimal pathways for the UK electricity sector transition to 2050. *Appl. Energy* **189**, 89–109 (2017).
24. Sasse, J.-P. & Trutnevyte, E. Distributional trade-offs between regionally equitable and cost-efficient allocation of renewable electricity generation. *Appl. Energy* **254**, 113724 (2019).
25. Trutnevyte, E., Stauffacher, M., Schlegel, M. & Scholz, R. W. Context-Specific Energy Strategies: Coupling Energy System Visions with Feasible Implementation Scenarios. *Environ. Sci. Technol.* **46**, 9240–9248 (2012).
26. DeCarolis, J. F. Using modeling to generate alternatives (MGA) to expand our thinking on energy futures. *Energy Econ.* **33**, 145–152 (2011).
27. Trutnevyte, E. EXPANSE methodology for evaluating the economic potential of renewable energy from an energy mix perspective. *Appl. Energy* **111**, 593–601 (2013).
28. Brown, T., Hörsch, J. & Schlachtberger, D. PyPSA: Python for Power System Analysis. *J. Open Res. Softw.* **6**, 4 (2018).
29. Brown, T., Schlachtberger, D., Kies, A., Schramm, S. & Greiner, M. Synergies of sector coupling and transmission reinforcement in a cost-optimised, highly renewable

European energy system. *Energy* **160**, 720–739 (2018).
